# Leveraging an Electronic Health Record Patient Portal to Help Patients Formulate Their Health Care Goals: Mixed Methods Evaluation of Pilot Interventions

**DOI:** 10.2196/56332

**Published:** 2024-08-29

**Authors:** Jody Naimark, Mary E Tinetti, Tom Delbanco, Zhiyong Dong, Kendall Harcourt, Jessica Esterson, Peter Charpentier, Jan Walker

**Affiliations:** 1 Department of Family Medicine Winchester Hospital Winchester, MA United States; 2 Section of Geriatrics Department of Internal Medicine Yale School of Medicine New Haven, CT United States; 3 Division of General Medicine Beth Israel Deaconess Medical Center Harvard Medical School Boston, MA United States; 4 CRI Web Tools LLC Durham, CT United States

**Keywords:** electronic pre-visit questionnaire, patient portal, multimorbidity, care priorities, goal, goals, priority, priorities, care plan, care plans, questionnaire, questionnaires, previsit, pre-visit, portal, portals, electronic health record, EHR, records, record, engagement, experience, experiences, attitude, attitudes, opinion, perception, perceptions, perspective, perspectives

## Abstract

**Background:**

Persons with multiple chronic conditions face complex medical regimens and clinicians may not focus on what matters most to these patients who vary widely in their health priorities. Patient Priorities Care is a facilitator-led process designed to identify patients’ priorities and align decision-making and care, but the need for a facilitator has limited its widespread adoption.

**Objective:**

The aims of this study are to design and test mechanisms for patients to complete a self-directed process for identifying priorities and providing their priorities to clinicians.

**Methods:**

The study involved patients of at least 65 years of age at 2 family medicine practices with 5 physicians each. We first tested 2 versions of an interactive website and asked patients to bring their results to their visit. We then tested an Epic previsit questionnaire derived from the website’s questions and included standard previsit materials. We completed postintervention phone interviews and an online survey with participating patients and collected informal feedback and conducted a focus group with participating physicians.

**Results:**

In the test of the first website version, 17.3% (35/202) of invited patients went to the website, 11.4% (23/202) completed all of the questions, 2.5% (5/202) brought results to their visits, and the median session time was 43.0 (IQR 28.0) minutes. Patients expressed confusion about bringing results to the visit. After clarifying that issue in the second version, 15.1% (32/212) of patients went to the website, 14.6% (31/212) completed the questions, 1.9% (4/212) brought results to the visit, and the median session time was 35.0 (IQR 35.0) minutes. In the test of the Epic questionnaire, 26.4% (198/750) of patients completed the questionnaire before at least 1 visit, and the median completion time was 14.0 (IQR 23.0) minutes. The 8 main questions were answered 62.9% (129/205) to 95.6% (196/205) of the time. Patients who completed questionnaires were younger than those who did not (72.3 vs 76.1 years) and were more likely to complete at least 1 of their other assigned questionnaires (99.5%, 197/198) than those who did not (10.3%, 57/552). A total of 140 of 198 (70.7%) patients responded to a survey, and 86 remembered completing the questionnaire; 78 (90.7%) did not remember having difficulty answering the questions and 57 (68.7%) agreed or somewhat agreed that it helped them and their clinicians to understand their priorities. Doctors noted that the sickest patients did not complete the questionnaire and that the discussion provided a good segue into end-of-life care.

**Conclusions:**

Embedding questionnaires assaying patient priorities into patient portals holds promise for expanding access to priorities-concordant care.

## Introduction

Persons with multiple chronic conditions face increasingly complex medical regimens. Particularly common among older adults, these involve many medications, frequent health care encounters with multiple clinicians, and an escalating number of health care tasks that are often burdensome and of unclear benefit [1,2]. Disease-specific guidelines do not address this compounded burden. Importantly, clinicians may not focus on what matters most to these patients who vary widely in their health priorities [3-5].

To address these issues, a national group of patients, clinicians, care partners, and others developed Patient Priorities Care (PPC), a process designed to identify and align decision-making and care with an individual’s health priorities [6]. Health priorities include those health outcome goals patients most desire, given health care they are willing and able to receive. When combined with decision-making that aligns patient and clinician priorities, studies indicate that identifying such goals is feasible for both patients and clinicians and effective at increasing priorities-concordant care [7-9].

Developed initially as a facilitator-led process, as efforts to spread PPC increased, an initial challenge emerged. Facilitator-led identification of priorities requires access to a trained member of a health care team, and this may not be feasible in many settings, limiting access to priorities-aligned care for many individuals who would benefit. In response to this challenge, the PPC team created a web-based, self-directed process to guide patients in identifying their own health priorities. Once developed, the next challenge was how to get this information to clinicians so it could inform decision-making and care.

To develop and test approaches, the PPC team partnered with “OurNotes” investigators who had implemented previsit questionnaires that pose broad questions, bring patients’ words directly into the record, and share patients’ answers with clinicians before or during visits [10,11]. Our primary aim was to develop new electronic mechanisms that could replace the facilitator, while still encouraging patient engagement and satisfaction with PPC. A secondary aim was to evaluate qualitatively primary care doctors’ perceptions of the pilots’ workflow and usefulness.

## Methods

### Overview

Following a “Plan Do Study Act (PDSA)” approach, we tested different ways for patients to identify their health priorities and convey them to their primary care clinicians. PDSA is an iterative process that originated in industry and has been widely applied to quality improvement in health care [12]. Implementers identify a desired change (Plan), test the change (Do), assess the effectiveness of the change (Study), and identify revisions for the next cycle (Act). We recruited 2 primary care practices in suburban Boston to participate in the pilot efforts. Part of the Beth Israel Lahey Healthcare (BILH) system, both use the Epic electronic health record (EHR) [13]. Eligible patients were those aged 65 years and older, who were registered on the MyLaheyChart patient portal. Among patients aged 65 years or older in the 2 practices, approximately 72% and 55% were on the portal.

### Ethical Considerations

The study was certified as exempt by the institutional review boards of Lahey Medical Center (Protocol #20213123) and Beth Israel Deaconess Medical Center (Protocol #2021P000327).

### Interventions

#### Intervention 1a: MyHealthPriorities Website

**Figure 1:**
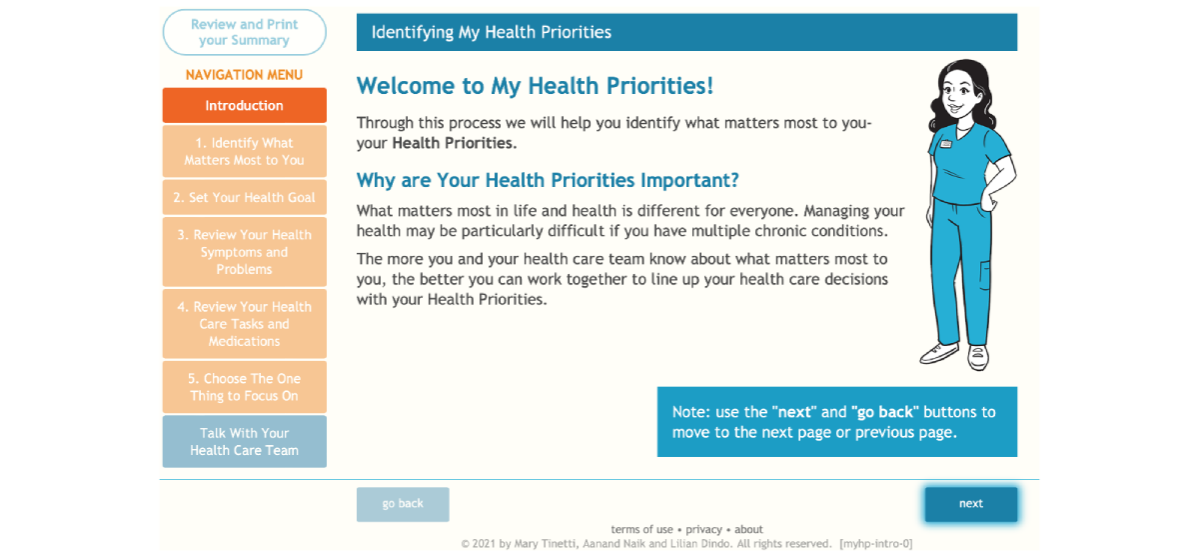
Screenshot from MyHealthPriorities website.

**Figure 2:**
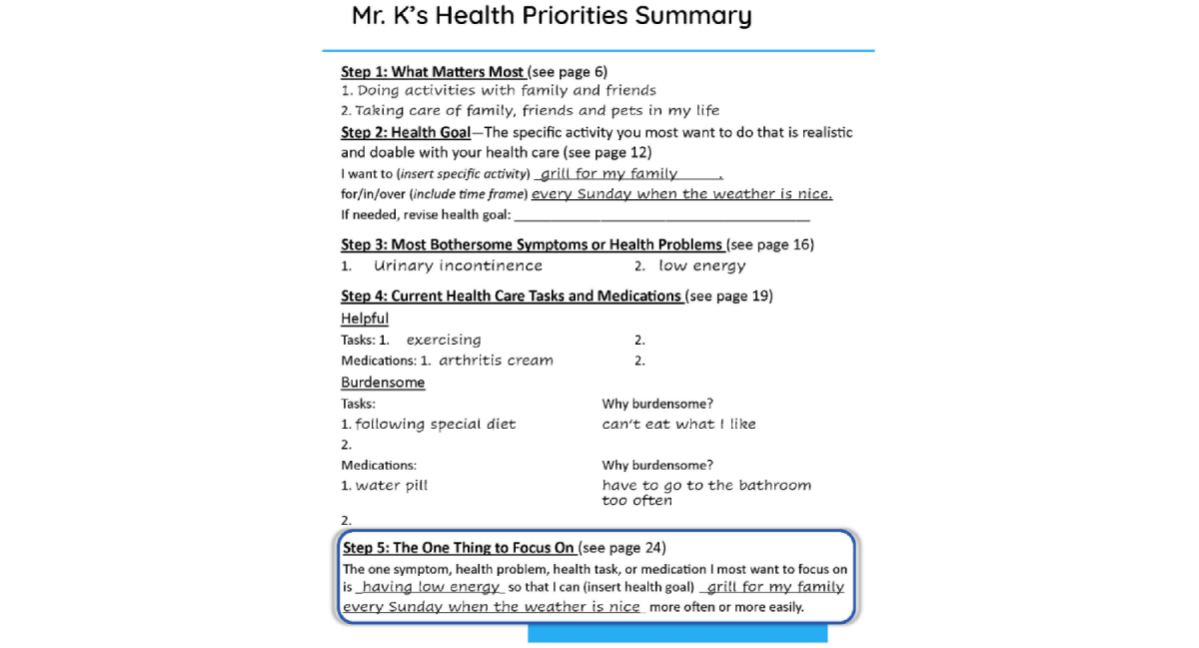
Sample Summary from MyHealthPriorities website.

We first invited patients to complete self-directed identification of health priorities at the MyHealthPriorities website [14] and to bring the resulting printout to their visit. MyHealthPriorities (MHP), developed through PPC, is a user-friendly, stand-alone site with visual cues, illustrations, prompts, and examples that leads patients through the PPC health priorities identification process (Figure 1). Patients could register, allowing them to update their answers in a later log in, or they could complete the identification process as a guest. As the patient proceeded through the website’s steps, the text was automatically self-populated as needed with answers from previous related questions. When patients completed the questions, the website displayed a 1-page Health Priorities Summary (Figure 2), with prompts asking them to print the Summary and bring it to the next appointment. They could also save a copy. The final website pages presented suggestions for talking with their clinicians about their health priorities and what matters most to them. For each patient, the website tracked the last page visited and the time spent on the site.

The 10 physicians from the 2 offices participated in a 1-hour training session designed to introduce them to PPC and the pilot. It included an overview and brief background of PPC, screenshots of the website, encouragement to look at it firsthand, a sample 1-page Summary that patients would bring to visits, and copies of the previsit communications sent to patients via the portal. The session did not include training about how to provide priorities-concordant care. We provided lists of the patients contacted and encouraged physicians to ask them about PPC if they did not bring it up during the visit.

The BILH study team ran periodic reports to identify eligible patients scheduled for nonemergency office visits with the practices’ 10 physicians. Among these patients, we asked their doctors to select patients by considering their burden of illness and potential benefit, and their likely willingness to complete the questionnaire. Approximately 2 weeks prior to their appointments, the team sent an email message to the selected patients directing them to an introductory letter in the patient portal; it explained the questionnaire and the study, that participation was voluntary, and that choosing not to participate would not affect their care at the practice. Approximately 1 week prior to the appointment, we sent a portal invitation letter with a unique patient-specific link to the MHP website; the link enabled us to track patients as they completed the interview and to contact them about their experiences with the website. When they brought summaries to the office, staff scanned and uploaded them to the “Media” section of the Epic EHR, where they were accessible to the care team.

#### Intervention 1b: Revised MHP Website

In our initial effort (intervention 1a), few patients went to the website and when we interviewed patients as part of the planned evaluation, several indicated they had not understood they needed to print the summaries or bring them to their appointments. Based on this feedback, we made some adjustments and conducted a second field period in 1 of the practices. We revised the website’s printing instructions and made the language in the invitation letter asking them to bring the Summary to the visit more prominent, and we convened the 5 physicians for a refresher training session. The study team ran periodic reports of upcoming appointments and asked the physicians to notify the team of any patients they wished to exclude. We sent the same link to all remaining patients (as opposed to the unique links in intervention 1a), which enabled more efficient mailings to patients, but also made website tracking anonymous and precluded follow-up with individual patients.

#### Intervention 2: Epic Previsit Questionnaire

As few patients went to the MHP website and even fewer printed their summaries or brought them to their appointments, we decided to test a different approach in the second practice: an Epic previsit questionnaire. This would allow patients to stay within Epic rather than going to an outside website, with results captured in Epic and automatically conveyed to clinicians. We created a questionnaire that distilled the MHP website into 8 items and mirrored the website’s response options. The Summary produced by Epic displayed both the text of the questions and the patient’s responses and was saved automatically when submitted. Clinicians could view the Summary in the “Schedule” view and in the “Synopsis” and “Questionnaires” sections of the EHR, and we created a dot phrase to enable them to insert the Summary text into the visit note. Patients could review the Summary in the patient portal.

In addition to the absence of a graphic presentation, the MHP website had 4 important characteristics that could not be replicated in the EHR questionnaire. First, the EHR did not have a self-population function that displayed previous answers. To compensate, we added text to relevant questions, for example, “Please review your Health Goal (#2 above) and the things you selected as most burdensome (#3, #4, and #5).” Second, for questions asking patients to select items from a list, the website sometimes displayed lists in multiple columns to enable patients to avoid scrolling. In contrast, the EHR questionnaire did not have column functionality, and respondents had to scroll to see all of the response options. Third, the website accepted a specified number of responses, such as for questions asking for “1 or 2” responses from a list. The EHR questionnaire allowed 1 response only, or “all that apply.” We, therefore, added instructions to the question text, for example, “We suggest you choose 1 or 2” as needed, and then saved all options selected even if they were more than “1 or 2.” Finally, in contrast to the website that offers prompts, examples, tips for talking with clinicians about their health priorities, and other explanatory material, we could not develop functionality that separated such material visually from the questions themselves. Unable to contrast instruction and exemplars by using different fonts or colors, we elected to not include any such material. The EHR questionnaire can be found in Multimedia Appendix 1.

All 5 physicians in the second practice were given a refresher presentation on the PPC project and a description of how the Epic questionnaire works. They were given a copy of the 8 questions on the questionnaire and the introductory letter the patients received. They were taught where to look in Epic for the answers to the questions and how to add the questions and answers to the progress note with a dot phrase (smart phrase) if they so desired. They were again asked to jot down if time allowed, any thoughts they had about the project as it progressed.

In the first week of intervention 2, the study team again sent an introductory letter to eligible patients scheduled for routine office visits with the practice’s 5 physicians, this time explaining that during the intervention period, they would receive the questionnaire 3 days before their appointments. Thereafter, the EHR automatically sent the questionnaire as part of each eligible patient’s previsit email 3 days before visits, including patients scheduled after the first week who did not receive the introductory letter.

After completing preregistration details for upcoming scheduled visits, patients in the practice were invited to complete from 1 to 5 questionnaires assigned routinely by the practice or system. These were followed by the study questionnaire, which came last. For each appointment, portal tracking included whether each study questionnaire’s individual items were answered, the submission status, the number of minutes between starting and submitting the questionnaire, and the number of other questionnaires sent to and submitted by the patient.

### Evaluation

#### Intervention 1a

We used tracking data to count patients who visited the website, to summarize their movement through the site, and to calculate the median and IQR for how much time they spent. We made 2 attempts by phone to interview patients who went to the site, using open-ended questions. We asked physicians to provide the investigators with informal written or verbal comments about their experiences.

#### Intervention 1b

We used tracking data to summarize patients’ movement through the website and calculated the median and IQR for time spent, and we asked physicians for informal written or verbal feedback from their conversations with patients.

#### Intervention 2

We developed an online survey of patients who had completed questions in a study questionnaire in intervention 2, and—anticipating that care partners might respond for some patients—we developed a slightly reworded version for care partners. We asked them about any perceived difficulty of the EHR questions, whether they discussed their answers with their clinicians, whether anything changed in their care, whether they thought their answers helped them and their clinicians to understand their priorities better, and about their preferences for completing the questionnaire. The survey also included a series of sociodemographic questions and free-text items about questions that they may have considered difficult, what was changed after discussion with the clinician, suggestions for improvement, and other comments. The sociodemographic and free-text questions were optional; all others required responses. We sent patients a portal invitation message with a unique link to the survey; nonrespondents received a reminder message about 10 days later. As an incentive, we offered a chance to win US $50 in a raffle if patients completed the survey. The surveys are included in Multimedia Appendices 2 and 3. The survey was conducted using Research Electronic Data Capture (REDCap; Vanderbilt University).

A secondary aim was to evaluate primary doctors’ perceptions of workflow and the usefulness of the information obtained from the EHR questionnaire. We conducted a 60-minute focus group with the participating doctors on Zoom (Zoom Video Communications). The discussion was led by a coinvestigator (TD) who is a health services researcher and a primary care doctor. We asked them about the usefulness of patients’ responses, the review process and its effect on workflow, and ideas for the future (discussion guide in Multimedia Appendix 4).

### Analysis

In interventions 1a and 1b, we used descriptive statistics to summarize patients’ completion of steps in the website interview and, by using website tracking data, the median and IQR of time they spent on the site. In intervention 2, we downloaded portal tracking and REDCap patient survey responses into SAS data sets (SAS Institute). We used descriptive statistics to summarize patient participation, including the proportion of visits with questionnaires completed or partially completed, the proportion of questions answered, the survey response rate, and the survey responses, and calculated the median and IQR for questionnaire completion time. We compared patients who did and did not complete the Epic questionnaire using chi-square tests on categorical variables and *t* tests on continuous variables.

We used the immersion-crystallization technique in iterative processes to analyze the qualitative data [15]. Three authors, including the facilitator (JN, TD, and JW), met immediately after the clinician focus group and identified the important themes each had heard. Once the discussion was transcribed, 2 authors (JN and JW) read the transcript, then met to review the original list of themes, reread the transcript together, and create a final set of themes. One author (JN) then selected representative comments. Similarly, 2 authors (JN and JW) independently reviewed free-text responses from the patient survey, then worked together to define the important topic areas and code the responses, discussing disagreements until they reached consensus. One of the authors (JW) then identified representative comments from each area.

## Results

A flowchart describing the interventions is displayed in Multimedia Appendix 5.

### Intervention 1a

Intervention 1a ran from December 7, 2021, to March 29, 2022. We sent 202 invitations to patients; 35 (17.3%) went to the MHP website, 33 (16.3%) went beyond the registration page, 23 (11.4%) completed all of the questions, 10 (5.0%) printed or saved their results, and 5 (2.5%) brought summaries to their appointments (Table 1). The median website session time for those who went past the registration page was 43.0 (IQR 28.0) minutes.

In 21 phone interviews, patients who went to the website generally viewed the website positively, calling it “a good idea” and “useful.” One person said it “made me think about what I’m not doing for myself.” Fewer viewed it negatively, most often because they thought it did not pertain to them, for example, it was for someone “more elderly.” A total of 5 patients described completing the questions but did not have a printer, did not realize the physician could not see their answers, or forgot to bring the printed Summary to the appointment. In informal comments jotted down after the visits, physicians for the 5 patients who brought in the Summary felt that it did not add value overall to the encounter.

**Table 1:** Interventions 1a and 1b: completion rates of self-directed priorities on the MyHealthPriorities website.

		Phase 1a, n (%)	Phase 1b, n (%)
Patients contacted		202 (100.0)	212 (100.0)
Went to website		35 (17.3)	32 (15.1)
**Section reached**			
	Identify what matters most to you	33 (16.3)	31 (14.6)
	Set your health goal	28 (13.9)	26 (12.3)
	Review your health symptoms and problems	28 (13.9)	23 (10.8)
	Review your health care tasks and medications	26 (12.9)	22 (10.4)
	Choose the one thing to focus on	23 (11.4)	19 (9.0)
	How to talk with your health care team	17 (8.4)	12 (5.7)
Printed or saved Summary		10 (5.0)	13 (6.1)
Brought Summary to appointment		5 (2.5)	4 (1.9)

### Intervention 1b

Intervention 1b ran from August 8, 2022, to October 7, 2022. We sent invitations to 212 patients; 32 (15.1%) went to the website, 31 (14.6%) went past the registration page, 19 (9.0%) finished the questions, 13 (6.1%) printed or saved the Summary, and 4 (1.9%) brought the summaries to their appointments with 3 clinicians (Table 1). Among the clinicians, 2 reported finding the summaries useful and 1 did not; 1 reported that a patient found it useful, in contrast to 3 who did not. Among patients who went past the registration page, the median session time was 35 (IQR 35) minutes.

### Intervention 2

Intervention 2 ran from September 14, 2022, to December 14, 2022. We sent the introductory letter to 521 patients who had appointments scheduled before the intervention launched. The EHR then included the questionnaire in all previsit messages to 891 patients (521+370 whose appointments were scheduled after launch) before 1139 scheduled routine visits (Multimedia Appendix 5). Among 750 patients who kept at least 1 visit during the intervention, 198 (26.4%) completed 205 questionnaires. They submitted 153 (77.3%) of them before visits with the 5 participating physicians; each physician received between 23 and 44 questionnaires. Among the remaining 52 completed questionnaires, 29 (14.6%) were completed and not submitted, 17 (8.6%) were submitted to nonparticipating nurse practitioners after a change in the originally scheduled appointment, and 6 (3.0%) were submitted before visits that were not kept. The median completion time for submitted questionnaires was 14.0 (IQR 23.0) minutes.

The average age of patients who completed or partially completed the questionnaire was 72.3 years; it was 76.1 years for those who did not (Table 2). Patients who completed questionnaires had fewer primary care, nonprimary care, and emergency visits than those who did not. Except for 1 patient, all who completed the study questionnaire also completed other assigned questionnaires; 10.3% (57/552) of those who did not complete a study questionnaire completed other assigned questionnaires. Differences between respondent and nonrespondent age, number of primary care and emergency visits, and completion of other questionnaires were significant.

Among the 205 completed questionnaires, the 8 main questions were answered 62.9% (129/205)-95.6% (196/205) of the time (Table 3). Items about what matters most, specifying a health goal, what interferes with achieving the health goal, and whether medications are burdensome were answered at least 90% of the time. Items about burdensome and helpful health care tasks and helpful medications were answered 72.7% (149/205)-89.8% (184/205) of the time. The question asking patients to specify the “One Thing that most interferes with achieving the Health Goal” was answered 62.9% (129/205) of the time.

**Table 2:** Intervention 2: characteristics of 750 patients who kept at least 1 appointment and received Epic previsit questionnaires.

			Completed or partially completed at least 1 Epic questionnaire (n=198)		Completed 0 Epic questionnaires (n=552)		*P* value	
**Age (years), n (%)**								<.001
	65-69	70 (35.4)		143 (25.9)				
	70-74	65 (32.8)		110 (19.9)				
	75-79	44 (22.2)		117 (21.2)				
	80-84	16 (8.1)		94 (17.0)				
	85+	3 (1.5)		88 (15.9)				
Average (years), mean (SD)			72.3 (5.5)		76.1 (7.6)		<.001	
**Sex, n (%)**								.36
	Female	124 (62.6)		325 (58.9)				
	Male	74 (37.4)		227 (41.1)				
**# visits last 12 months, mean (SD)**								
	Primary care	3.92 (3.8)		5.03 (4.8)		<.01		
	Nonprimary care	2.61 (5.5)		3.14 (6.3)		.26		
	Emergency	0.64 (1.9)		1.13 (2.4)		<.01		
**Completed at least 1 other assigned questionnaire, n (%)**								<.001
	Yes	197 (99.5)		57 (10.3)				
	No	1 (0.5)		495 (89.7)				

**Table 3:** Intervention 2: responses to questions in 205 submitted Epic previsit questionnaires.

		Values, n (%)
**1. Identify what matters most to you in your life and health. Think about what gives your life meaning, purpose, joy, or satisfaction. Select items from the following that matter most to you right now. We suggest you select up to 3.**		196 (95.6)
	(If other) Please explain (free text).	3 (1.5)
2. Based on what you selected above as what matters most to you, what specific activity would you like to be able to do now? This activity should be one that you think is realistic to achieve, with help from your health care team. This activity is your health goal (free text).		188 (91.7)
**3. You may have several bothersome symptoms or health problems. Which ones most interfere with achieving your health goal? We suggest you choose 1 or 2.**		188 (91.7)
	(If other) Please explain (free text).	24 (11.7)
**4. It is helpful for your clinicians to know what health care tasks you find difficult or burdensome. Below is a list of common health care tasks. You may do some of them regularly. Which ones do you find the most burdensome or not helpful? We suggest you select 1 or 2.**		184 (89.8)
	(If other) Please explain (free text).	22 (10.7)
**5a. Now please think about your medications. Do any of them cause problems such as dizziness, tiredness, or confusion, or do they cost too much? Does the discomfort they cause outweigh any positive effect?**		195 (95.1)
	Please enter the name of a medication you find most burdensome or not helpful (free text).	20 (9.8)
	What makes the medication burdensome? (free text).	19 (9.3)
**5b. Do you have any other medications you find burdensome?**		19 (9.3)
	(If yes) Please specify the name of an additional medication you find most burdensome or not helpful (free text).	0 (0)
	What makes the medication burdensome? (free text).	0 (0)
6. Please review your health goal (#2 above) and the things you selected as most burdensome (#3, #4, and #5). Among the burdensome symptoms, tasks, and medications you selected, which 1 thing most interferes with achieving your health goal? (free text)		129 (62.9)
**7. It is also important for your health care team to know what health care tasks you find helpful. Please review the list of common health care tasks again. This time, select those you find most helpful. We suggest you select 1 or 2.**		181 (88.3)
	(If other) Please explain (free text).	7 (3.4)
**8. It is also important for your health care team to know what medications you find helpful. Please think about your medications again and specify the name of a medication you find most helpful (free text).**		149 (72.7)
	Do you have any other medications that you find helpful?	139 (67.8)
	(If yes) Please enter the name of another medication you find most helpful (free text).	52 (25.4)

### Patient Survey

We received 140 responses from 198 patients, a response rate of 70.7%. Among these, 54 respondents did not recall completing the priorities questionnaire and exited the survey.

Among the 86 respondents who remembered completing the questionnaire, all identified themselves as patients (Table 4). Nearly all spoke English (95.3%, 82/86), 93.0% (80/86) were White, 58.1% (50/86) were female, 61.7% (53/86) were younger than 75 years, and 45.3% (39/86) had completed a college degree. A total of 12 (14.0%) reported having 3 or more chronic illnesses, 16.3% (14/86) reported being in fair or poor health, and 15.1% (13/86) reported 3 or more visits to primary care physicians in the last 12 months.

Three-quarters of the patients (76.7%, 66/86) reported completing 1 priorities questionnaire, most (82.6%, 71/86) did not use the portal to look at the Summary again after they submitted it, and most (90.7%, 78/86) did not remember having difficulty completing it (Table 5). A total of 8 patients (9.3%) found some items difficult to answer, most often the questions about their specific health goals, which life activities matter most to them, and which health care tasks they find most burdensome. They wrote that they found such questions confusing, for example, “I am not sure what you mean by some of the questions,” or that they did not feel prepared to answer, for example, “[I’m] just not sure of the most important things right now.”

More than a third of the respondents (33.7%, 29/86) reported having discussed their answers with the clinician during the visit; 3.5% (3/86) reported discussion followed by a change in care and 30.2% (26/86) reported discussion with no change. In free-text descriptions of discussions and changes, 10 patients cited specific topics such as weight loss, blood pressure, and medication changes, and 6 comments described conversations that were more general.

Two-thirds (66.3%, 57/86) agreed or somewhat agreed that completing the questionnaire helped them and that their clinicians understand what is most important in their health and care. They most often wanted to update their answers once a year (82.5%, 47/57) and to have more than 3 days to do so (79.0%, (45/57).

Patients wrote 54 free-text responses to the question asking for suggestions for improvement. The most common response described the questionnaire as “easy to follow,” “fine” as is, or suggested “no changes” (26 responses), 3 expressed appreciation for its thought-provoking questions, and 3 persons preferred talking with their providers rather than completing questionnaires. A total of 4 respondents made suggestions; 2 related to questionnaire response options, 1 suggested dropping the questionnaire since “it doesn’t seem like anyone pays attention to them,” and 1 suggested developing a way for patients to keep track of the questions they want to ask in an appointment. Three respondents wanted more time to complete the questionnaire.

Of 18 “other comments,” the most common were complimentary comments about providers (8 comments) and 2 characterized the questionnaire as a good idea.

**Table 4:** Intervention 2: self-reported characteristics of 86 patient respondents to a survey about their experience with the Epic previsit questionnaire.

		Values, n (%)
**Respondent**		
	Patient	86 (100.0)
	Care partner	0 (0.0)
**Age (years)**		
	65-69	28 (32.6)
	70-74	25 (29.1)
	75-79	25 (29.1)
	80+	4 (4.7)
	Missing	4 (4.7)
**Sex**		
	Female	50 (58.1)
	Male	32 (37.2)
	Missing	4 (4.7)
**Race** **(check all that apply)**		
	White	80 (93.0)
	American Indian or Pacific Native	1 (1.2)
	Asian	1 (1.2)
	Other	2 (2.3)
	Missing	4 (4.7)
**Education**		
	High school or less	21 (24.4)
	Some college or technical school	22 (25.6)
	4-year college or some grad school	21 (24.4)
	Masters or doctoral degree	18 (20.9)
	Missing	4 (4.7)
**Self-reported health status**		
	Excellent	6 (6.9)
	Very good	30 (34.9)
	Good	32 (37.2)
	Fair	12 (14.0)
	Poor	2 (2.3)
	Missing	4 (4.7)
**Number of chronic illnesses**		
	0	21 (24.4)
	1-2	49 (57.0)
	3 or more	12 (14.0)
	Missing	4 (4.7)
**Visits with PCP^a^ in last 12 months**		
	1-2	70 (81.4)
	3 or more	13 (15.1)
	Missing	3 (3.5)
**Language at home**		
	English	82 (95.3)
	Other	1 (1.2)
	Missing	3 (3.5)
**Hispanic or Latinx ethnicity**		
	Yes	0 (0.0)
	No	83 (96.5)
	Missing	3 (3.5)

^a^PCP: primary care physician.

**Table 5:** Intervention 2: responses from 86 patient survey respondents about their experiences with the Epic previsit questionnaire.

		Values, n (%)
**How many times did you complete the questionnaire?**		
	Once	66 (76.7)
	More than once	20 (23.3)
**Once it was submitted, did you ever look again at your answers using MyChart?**		
	Yes	13 (15.1)
	Tried, but couldn’t find it	2 (2.3)
	No	71 (82.6)
**Did you find the questions difficult to answer?**		
	No, don’t remember having difficulty	78 (90.7)
	Yes, some were difficult	8 (9.3)
**Which questions were difficult?**		
	What matters most	4 (50.0)
	Activity I want to do or health goal	5 (62.5)
	Which symptoms or problems are bothersome	2 (25.0)
	Which tasks are burdensome	3 (37.5)
	Which medications are burdensome	1 (12.5)
	Task or medication that interferes with health goal most	1 (12.5)
	Which tasks are helpful	2 (25.0)
	Which medications are helpful	1 (12.5)
**Did you discuss your answers with your provider in the visit?**		
	Yes, discussed and changed something	3 (3.5)
	Yes, discussed, did not change anything	26 (30.2)
	No, did not discuss	28 (32.6)
	Do not know or not sure	26 (30.2)
	Missing	3 (3.5)
**In general, completing the questionnaire helped me and my clinicians understand what matters to me^a^**		
	Agree	38 (44.2)
	Somewhat agree	19 (22.1)
	Somewhat disagree	6 (7.0)
	Disagree	5 (5.8)
	Do not know or not sure	15 (17.4)
	Missing	3 (3.5)
**How often would you like to update your answers?**		
	Once a year	47 (82.5)
	More than once per year	1 (1.7)
	Never, do not need to update	9 (15.8)
**When would you like to update?**		
	3 days before visit	12 (21.1)
	4-7 days before	34 (59.7)
	>7 days before	11 (19.3)

^a^Respondents answering disagree, somewhat disagree, or do not know or not sure skipped the next 2 questions.

### Physician Focus Group

Overall, participating physicians were concerned about inefficiencies and other effects on workflow, and they described both positive and negative impressions of their patients’ submissions. They reported that none of their patients had brought up the questionnaire during the visit, thereby creating work by requiring the clinician to remember to look for it. Depending on the EHR view they used, some clinicians found it difficult to find the priorities questionnaire. They felt that integrating it well into their workflow would require notification that the questionnaire had been submitted, easy access to the patient’s responses, and—having forgotten about the project’s dot phrase—a mechanism to import responses into the visit note. They felt the current workflow would be too cumbersome to implement for all Medicare patients.

They described the priorities responses as “a good gestalt” about patients’ views and, in particular, a “good segue” into end-of-life discussions. Except for when they discussed such decisions, they reported the conversations did not affect their visit notes. All agreed there were “no surprises” in any of their conversations. The responses they found most useful were those about burdensome medications. They noted that many of their patients who are older do not have computers and that those who did the questionnaire seemed to be among their younger and healthier patients, rather than their older and sicker patients who might have benefited more.

They described mixed responses from patients about the questionnaire, estimating about half thought it helpful and half thought it not helpful. They reported that some patients described being confused by being asked to finish a simple COVID-19 questionnaire, for example, followed by moving directly to questions about their life priorities.

After looking at the questionnaire itself during the discussion, the clinicians made suggestions about the wording of some questions and responses and thought some questions might be too similar to one another for some older patients to do independently. They suggested also it be presented to patients in conjunction with annual visits encouraged and funded by Medicare.

## Discussion

### Principal Findings

The principal goal of our inquiry was to pilot-test interventions designed to elicit patient priorities vital to the health and health care of older patients with chronic illness in primary care practices, without depending on a facilitator-mediated process. In spite of disappointments that were primarily procedural, patients reported feeling the online process worthwhile, and some reported it triggering introspective thinking. Some physicians found the questionnaire useful for feedback, particularly about medications and as a trigger for discussions about decisions concerning the end of life and health maintenance.

Though patients in intervention 1a reported liking a user-friendly interactive website developed by PPC, few clicked the link in the portal invitation to go to the site, and even fewer completed the steps necessary—including printing a Summary and bringing it to the appointment to discuss their answers with their doctors. Intervention 1b added instructions and website changes based on patient feedback, but completion rates did not improve. In Intervention 2, a far higher proportion of patients completed a much-reduced version distilled into an EHR questionnaire that was included in a practice’s previsit materials for routine office visits with patients aged 65 years or older. The primary care doctors participating reported favorably on varied aspects of the resulting conversations they had with patients. However, they agreed that effective, wider implementation would necessitate implementing seamless workflows related to (1) clinician notifications about the questionnaire and (2) how to include selected parts of the patient’s report in the visit note.

Health systems are experimenting with using patient portals to collect information ahead of visits [16-21]. Making the information visible in the EHR as part of the clinician workflow may save time in visits. These protocols often use questionnaires and sometimes include links encouraging patients to view external educational websites, and 1 tested a patient-computer dialog (rather than a questionnaire) to take a family health history. Our pilot incorporated many of these elements in 2 different approaches testing the feasibility of replacing a facilitated conversation that takes place outside of visits. While our work may eventually make visits more efficient, its chief objective is to enable patient-clinician collaboration leading to streamlined care that aligns with patients’ overall health priorities.

Our initial PDSA cycles, phases 1a and 1b, tested a stand-alone portal invitation to an in-depth, interactive website questionnaire (as opposed to a read-only educational site) with no direct link to capture results in the EHR; we depended on patients to deliver the results to their clinicians. When that process failed, we used the portal’s native questionnaire capabilities in phase 2 to build a relatively bare-bones questionnaire whose results would be automatically captured in the EHR, and, instead of sending a stand-alone message, included the invitation in the practice’s standard previsit messaging protocol. Both approaches had strengths and weaknesses. While patients had few problems with the free-standing website and found it “interesting,” few actually went to the site, and even fewer completed all the steps necessary, from answering all of the questions to bringing a printed Summary to the visit. In contrast, far more patients completed the EHR questionnaire as part of their practice’s standard previsit request for information. In fact, nearly all patients who completed previsit tasks completed all requested questionnaires, which holds promise for embedding into portals questionnaires addressing patient priorities.

Doctors reported some clinically important effects, but they also found the intervention inefficient; the older, sicker patients who stood to benefit the most did not submit the questionnaire, the doctors reported finding “no surprises” in patients’ responses, and the questionnaire workflow was cumbersome. As the aim of this study was to evaluate the feasibility of patients identifying their health priorities without guidance from health care team members, we did not educate the doctors about how to provide priorities-concordant care. It is not surprising, therefore, that clinicians did not find the information useful and that there were few changes in care. Previous and ongoing studies support the effectiveness of patient priorities aligned care in improving clinical outcomes [22,23]. These results also suggest it is important to prepare both patients and clinicians to identify and act on health priorities.

### Limitations

We do not have information on why patients did not go to the website. Data about whether the invitation was opened were not available. We lacked demographic and other data on participants enrolled in interventions 1a and 1b and cannot comment on whether there were differences between those who did and did not access the website or complete the identification of priorities. Among those who did access the website, we do not know who registered and who visited as a guest. We did not have information about patients’ access to computer equipment or about their computer skills. We did not compare the quality of patients’ responses to the in-depth website version to the responses in the shorter Epic version.

### Implications for Future Work

We encountered many obstacles and the PDSA process was effective in helping us to identify problems and make relevant adaptations. Our results point to the next steps in our aim to incorporate self-identified health priorities into clinical workflow. The greater completion rate using the Epic questionnaire highlights the benefits of embedding previsit forms in the patient portal. Even so, the relatively modest uptake underscores a need for strategies that encourage and facilitate greater patient participation.

Beyond improved completion of previsit forms, questionnaires need to offer greater interactivity and adaptability. Probes, tips, scripts, examples, and the capacity to carry forward responses are a few examples of features available on the MyHealthPriorities website that were lost in the EHR questionnaire. Recent developments that allow external inputs into EHRs through apps available to patients hold promise for combining the convenience of EHR-embedded material with the interactivity and adaptability needed to capture important patient-generated data, such as their health priorities. Such innovations face challenges such as vulnerability to hacking, introduction of digital viruses, onerous business associate agreements, and faulty conclusions from AI apps. Moreover, methods for handling inevitable updates will need to be developed, either centrally by app developers or locally, with each site maintaining its own expertise. In the short term, there needs to be a readily accessible place that can receive material like the PPC Summary and distribute it into the EHR for clinician access and evaluation.

While incorporating the interactive MHP website into the patient portal will likely increase uptake, it will not address the disparity in completion rates among younger and healthier individuals compared with older adults with greater morbidity who are those most likely to benefit from priorities-concordant care [24]. In a recent study, we found users to be healthier, better educated, and more likely White than the general older adult population [24]. Access to, and familiarity with computers also remain as barriers for older and minority populations [25-27]. Computer access can be addressed, for example, by kiosks in public locations or health facilities. While some individuals may never be able to use digital tools, others who are unable or unwilling to complete the web-based priorities identification alone may benefit from guidance from family, other care partners, or members of the health care team. We anticipate that new and varied options will expand access to health priorities identification—and to patient priorities aligned care to a broader population who could benefit.

## Appendix

Multimedia Appendix 1 Epic previsit priorities questionnaire.

Multimedia Appendix 2 Patient postintervention survey.

Multimedia Appendix 3 Care partner postintervention survey.

Multimedia Appendix 4 Clinician focus group discussion guide.

Multimedia Appendix 5 Interventions flowchart.

## Abbreviations

BILH: Beth Israel Lahey Healthcare
EHR: electronic health record
MHP: MyHealthPriorities
PDSA: Plan Do Study Act
PPC: Patient Priorities Care
REDCap: Research Electronic Data Capture
Summary: Health Priorities Summary
