# Supplementary material for: Leveraging an Electronic Health Record Patient Portal to Help Patients Formulate Their Health Care Goals: Mixed Methods Evaluation of Pilot Interventions
Source: JMIR Form Res. 2024 Aug 29;8:e56332. doi: 10.2196/56332 (PMC11393498; doi:10.2196/56332)
Supplement: Multimedia Appendix 1 [file formative_v8i1e56332_app1.doc]

**My Health Priorities – Epic Questionnaire**

**Your Health Priorities**

These questions will help you identify your health priorities- what matters most to you. Health priorities include the health and life goals and activities you want your health care team to help you with, given what you are willing and able to do.

You will be guided through a few questions. When finished, you will have a summary of your health priorities and the One Thing you most want to focus on. This will be shared with your health care team.

Sharing your answers with your health care team will help you make decisions that:

1. Line up with what matters most in your life and health.
2. Are consistent with the health care tasks and medications you are willing and able to do/take.

You can work on this on your own or have a family member or friend help.

**Step 1: Identify What matters most to you in your life and health:**

Think about what gives your life meaning, purpose, joy, or satisfaction. Choose **up to 3** of the following that matter most to you right now: [requires at least 1 response]

- Doing activities with family and friends
- Taking care of family and friends and/or pets in my life
- Participating in activities that support my religious or spiritual life
- Doing productive work (e.g., a job or volunteering)
- Taking care of myself
- Taking care of my home
- Walking or moving inside and outside of my home
- Choosing comfort and function over living longer
- Living as long as possible even if I am unable to do the things that are most important to me
- Living as long as possible, even if that means more treatments, pain, or discomfort
- Reducing the amount of time I devote to health care
- Avoiding spending time in the hospital
- Other, please explain: [pop-up free text field] ___________________

**Step 2: Set Your Health Goal**

Based on what you checked above as what matters most to you, what specific activity would you like to be able to do now? This specific activity is your Health Goal, and one that you think is realistic to achieve, with help from your healthcare team.

Here are some examples from other patients:

- I want to be able to have my friends over once a week for lunch and poker.
- I want to be able to walk 2 blocks in my neighborhood.
- I want to be able to travel by plane to attend my grandson’s wedding next summer.

Please write your Health Goal here: [free text field- required]

**Step 3: Identify Bothersome Symptoms/Problems:**

1. You may have several bothersome symptoms or health problems; of these, pick the 1 or 2 that you think most interfere with achieving the Health Goal you wrote above: [requires at least 1 response]

- Poor eyesight
- Poor hearing
- Leg swelling
- Trouble breathing (short of breath)
- Health care tasks that take too much time
- Urinary incontinence
- Having to go to the bathroom often
- Diarrhea
- Constipation
- Upset stomach/nausea
- Trouble sleeping
- Muscle weakness
- Pain
- Unsteadiness/trouble balancing or walking
- Feeling worried/nervous/anxious
- Feeling sad
- Feeling irritable
- Problems from treatments
- Dizziness
- Tiredness/lacking energy
- Other bothersome symptom or health problem (please explain): [pop-up free text field] ___________________

**Step 4: Identify Burdensome tasks and medications**

It is helpful for your clinicians to know what healthcare tasks you find difficult or burdensome. Here is a list of common health care tasks. You may do some of them regularly. Please mark 1 or 2 that you find most burdensome, or that are not helpful. [requires at least 1 response]

- Following a special diet
- Exercising
- Checking health signs, like weight or blood pressure
- Checking blood sugar
- Wearing a CPAP mask
- Wearing oxygen
- Using a cane or walker
- Having blood tests done
- Having x-rays done
- Going through other diagnostic tests, like mammography
- Having procedures done, like colonoscopy
- Visiting your primary care clinician
- Visiting specialists, like a cardiologist, pulmonologist, or urologist
- Visiting a counselor, like a psychologist or social worker
- Doing rehabilitation, like physical therapy or cardiac rehab
- Receiving in-home help
- Using transportation services
- Getting meal delivery
- Attending a community-based program such as senior day center, senior center, or support group
- Other (please explain): [pop-up free text field] ___________________

**Burdensome medications**

Now please think about your medications. Ask yourself, do any of them cause problems such as dizziness, tiredness, or confusion, or do they cost too much? Does the discomfort they cause outweigh any positive effect?

If yes, please enter the name of 1 or 2 medications you find most burdensome or not helpful. If you don’t remember the name or it is too long, you may write what it is used for. [responses optional]

Medication #1: ___________________________

What makes it burdensome? [pop-up free text field] ____________________

Medication #2: ___________________________

What makes it burdensome? [pop-up free text field] ____________________

**Step 5: Identifying the one thing you want to focus on first.**

In your answers above, you identified:

**The specific and realistic activity** that is your most important **Health Goal** you would like to be able to achieve.

Knowing the **Health Goal** you want to achieve and the problem most interfering with achieving this goal helps you and your healthcare team make decisions that align with what matters most to you. Focusing on this one thing doesn't mean your health care team won't address other problems you've identified; it's just a way to get started.

- Please **look back** at your Health Goal in **Step 2** and enter it here: [response required]

**HEALTH GOAL:** ________________________________

- Please **look back** at the items you marked as bothersome or burdensome in **Steps 3 and** **4**. While all of these may be bothersome and interfere with achieving your health goal, which **one** is **most bothersome** and **most interferes** with achieving your Health Goal? Please enter it here: [response required]

**MOST BOTHERSOME TASK OR MEDICATION:** _____________________________________

**Step 6: Additional Questions-Helpful Tasks and Medications.** It is also important for your health care team to know what healthcare tasks and medications you find helpful**.**

- Please review this list of common health care tasks again. **This time**, mark 1 or 2 that you find most **helpful**. [response required]
- Following a special diet
- Exercising
- Checking health signs, like weight or blood pressure
- Checking blood sugar
- Wearing a CPAP mask
- Wearing oxygen
- Using a cane or walker
- Having blood tests done
- Having x-rays done
- Going through other diagnostic tests, like mammography
- Having procedures done, like colonoscopy
- Visiting your primary care clinician
- Visiting specialists, like a cardiologist, pulmonologist, or urologist
- Visiting a counselor, like a psychologist or social worker
- Doing rehabilitation, like physical therapy or cardiac rehab
- Receiving in-home help
- Using transportation services
- Getting meal delivery
- Attending a community-based program such as senior day center, senior center, support group
- Other (please explain): [pop-up free text field] ___________________
- Please think about your medications again. **This time**, write the name of 1 or 2 medications you find most **helpful**. If you don’t remember the name or it is too long, you may write what it is used for. [responses optional]

Medication #1: ___________________________

Medication #2: _________________________

Thank you for answering these questions.

**Tips for Talking with Your Health Care Team -** [**https://patientprioritiescare.org/wp-content/uploads/2021/09/Pt-Tips_Talk-with-your-healthcare-team_9-28-21.pdf**](https://patientprioritiescare.org/wp-content/uploads/2021/09/Pt-Tips_Talk-with-your-healthcare-team_9-28-21.pdf)

When you are finished, click submit below.

**Thank you for participating in My Health Priorities!**
