# Supplementary material for: Leveraging an Electronic Health Record Patient Portal to Help Patients Formulate Their Health Care Goals: Mixed Methods Evaluation of Pilot Interventions
Source: JMIR Form Res. 2024 Aug 29;8:e56332. doi: 10.2196/56332 (PMC11393498; doi:10.2196/56332)
Supplement: Multimedia Appendix 2 [file formative_v8i1e56332_app2.doc]

PPC pilot at BILH: Patient Survey

12.21.22

*Free text and demographic items optional, answers to all others are required to advance through the survey. REDCap saves all responses, even if the patient drops out before finishing.*

1. In the last 3 months, how many times did you complete your Health Priorities questionnaire before a visit? [SHOW SCREENSHOT]
   1. I don’t remember completing the Health Priorities questionnaire 🡪 thank you and close
   2. Once
   3. More than once
2. Who is completing this survey?
   1. I am the patient
   2. I am someone who helps the patient, such as a family member or friend 🡪 care partner version of survey
3. After you submitted the Health Priorities questionnaire, did you ever look again at your answers by using MyChart?

a. No

b. Yes

c. I wanted to look but I couldn’t find the Health Priorities Online Summary

1. Please think about filling out the Health Priorities questionnaire. Did you find the questions difficult to answer?

a. No, I don’t remember having difficulty with the questions. 🡪 SKIP next 2 Qs

b. Yes, some questions were difficult to answer.

1. Which questions did you find difficult to answer? Check all that apply.
   1. Think about what gives your life meaning, joy, purpose, or satisfaction. What matters most to you right now?
   2. Based on what matters most to you, what specific activity would you like to be able to do now? This is your Health Goal.
   3. What bothersome symptom or health problem most interferes with achieving your Health Goal?
   4. What healthcare tasks do you find most burdensome or not helpful?
   5. What medications do you find most burdensome or not helpful?
   6. Which burdensome task or medication MOST interferes with achieving your Health Goal?
   7. Which healthcare tasks are most helpful in achieving your Health Goal?
   8. Which medications are most helpful in achieving your Health Goal?
2. Please describe what made the question(s) difficult to answer. We appreciate your feedback.

No word limit

1. During your visit, did you and your provider discuss your answers to the Health Priorities questionnaire?

a. No, we did not discuss the answers 🡪 skip next Q

b. Yes, we discussed the answers, but we didn’t change anything about my care

c. Yes, we discussed the answers and changed something about my care

d. Don’t know/not sure 🡪 skip next Q

1. Please describe what you and your provider discussed or changed, following the discussion of your Health Priorities.

No word limit

1. In general, completing the Health Priorities questionnaire helped me and my clincians understand what matters to me about my health and healthcare.
2. Disagree 🡪 skip next 2 questions
3. Somewhat disagree 🡪 skip next 2 questions
4. Somewhat agree
5. Agree
6. Don’t know/not sure 🡪 skip next 2 questions
7. How often would you like to update your Health Priorities questionnaire?
   1. Never, I don’t need to update it
   2. Once a year
   3. More often than once a year
8. When would you like to receive the request for completing your Health Priorities questionnaire?
9. 3 days before the visit
10. 4-7 days before the visit
11. More than 7 days before the visit
12. What improvements would you suggest for the future?

*For example: Was the questionnaire easy to follow? If the questions were hard to answer, what would make it easier? Did you have enough time before your appointment to consider your answers?*

No word limit

*The following demographic items are optional, the patient can skip them.*

1. How many visits did you have with your primary care provider in the last 12 months?
   1. 1-2
   2. 3 or more
2. In general, how would you rate your overall health?
   1. Excellent
   2. Very good
   3. Good
   4. Fair
   5. Poor
3. Do you have a chronic illness such as asthma, diabetes, COPD, high blood pressure, arthritis, heart disease, or cancer?
   1. Yes
   2. No 🡪 skip next Q
4. How many chronic illnesses do you have?
   1. 1 or 2
   2. 3 or more
5. Your age
   1. 65-69
   2. 70-74
   3. 75-79
   4. 80+
6. How do you describe yourself?
   1. Male
   2. Female
   3. Non-binary / third gender
   4. Prefer to self-describe ________
   5. Prefer not to say
7. What is the highest grade or level of school that you have completed?

a. High school or less

b. Some college or technical school

c. 4-year college degree or some graduate school

d. Masters or doctoral degree

1. What language(s) do you usually speak at home? Check all that apply.

a. English

b. Spanish

c. Other

1. Are you of Spanish/Hispanic/Latinx ethnicity?
   1. Yes
   2. No
2. What do you consider to be your racial background? Check all that apply.
   1. American Indian or Pacific Native
   2. Asian
   3. Black or African American
   4. Native Hawaiian or Pacific Islander
   5. White
   6. Other
3. Please write any other comments you would like to make. We truly appreciate your honest feedback.

No word limit

24. This is a research project, and we may want to interview a few patients by phone. If you would be willing to speak further with one of the researchers about the Health Priorities questionnaire, please write your Name and phone number or email address below.

No word limit

Submit button

Your feedback is important.

Thank you very much for answering these questions.
