# Supplementary material for: Leveraging an Electronic Health Record Patient Portal to Help Patients Formulate Their Health Care Goals: Mixed Methods Evaluation of Pilot Interventions
Source: JMIR Form Res. 2024 Aug 29;8:e56332. doi: 10.2196/56332 (PMC11393498; doi:10.2196/56332)
Supplement: Multimedia Appendix 3 [file formative_v8i1e56332_app3.doc]

PPC pilot at BILH: Patient Survey

Care Partner version

12.21.22

- *We will contact patients; if the respondent identifies as a care partner, s/he will be diverted automatically to this care partner version with the same questions asking about “the patient” or “you/the patient” rather than “you.”*
- *Free text and demographic items optional, answers to all others are required to advance through the survey. REDCap saves all responses, even if the patient drops out before finishing.*

1. In the last 3 months, how many times did you complete your Health Priorities questionnaire before a visit? [SHOW SCREENSHOT]
   1. I don’t remember completing the Health Priorities questionnaire 🡪 thank you and close
   2. Once
   3. More than once
2. Who is completing this survey?
   1. I am the patient
   2. I am someone who helps the patient, such as a family member or friend 🡪 care partner version of survey
3. After you/the patient submitted the Health Priorities questionnaire, did you/the patient ever look again at your answers by using MyChart?

a. No

b. Yes

c. I/the patient wanted to look but couldn’t find the Health Priorities Online Summary

1. Please think about filling out the Health Priorities questionnaire. Did you/the patient find the questions difficult to answer?

a. No, I don’t remember having difficulty with the questions. 🡪 SKIP next 2 Qs

b. Yes, some questions were difficult to answer.

1. Which questions did you/the patient find difficult to answer? Check all that apply.
   1. Think about what gives your life meaning, joy, purpose, or satisfaction. What matters most to you right now?
   2. Based on what matters most to you, what specific activity would you like to be able to do now? This is your Health Goal.
   3. What bothersome symptom or health problem most interferes with achieving your Health Goal?
   4. What healthcare tasks do you find most burdensome or not helpful?
   5. What medications do you find most burdensome or not helpful?
   6. Which burdensome task or medication MOST interferes with achieving your Health Goal?
   7. Which healthcare tasks are most helpful in achieving your Health Goal?
   8. Which medications are most helpful in achieving your Health Goal?
2. Please describe what made the question(s) difficult to answer. We appreciate your feedback.

No word limit

1. During the visit, did you/the patient discuss your answers to the Health Priorities questionnaire with the provider?

a. No, we did not discuss the answers 🡪 skip next Q

b. Yes, the answers were discussed, but nothing was changed about the patient’s care

c. Yes, the answers were discussed, and something was changed about the patient’s care

d. Don’t know/not sure 🡪 skip next Q

1. Please describe what was discussed or changed, following the discussion of the patient’s Health Priorities.

No word limit

1. In general, completing the Health Priorities questionnaire helped the patient and clincians understand what matters to the patient about health and healthcare.
2. Disagree 🡪 skip next 2 questions
3. Somewhat disagree 🡪 skip next 2 questions
4. Somewhat agree
5. Agree
6. Don’t know/not sure 🡪 skip next 2 questions
7. How often would you/the patient like to update the Health Priorities questionnaire?
   1. Never, I /the patient don’t need to update it
   2. Once a year
   3. More often than once a year
8. When would you/the patient like to receive the request for completing the Health Priorities questionnaire?
9. 3 days before the visit
10. 4-7 days before the visit
11. More than 7 days before the visit
12. What improvements would you suggest for the future?

*For example: Was the questionnaire easy to follow? If the questions were hard to answer, what would make it easier? Did you have enough time before the appointment to consider your answers?*

No word limit

*The following demographic items are optional, the respondent can skip them.*

1. How many visits did the patient have with his/her primary care provider in the last 12 months?
   1. 1-2
   2. 3 or more
2. In general, how would you rate the patient’s overall health?
   1. Excellent
   2. Very good
   3. Good
   4. Fair
   5. Poor
3. Does the patient have a chronic illness such as asthma, diabetes, COPD, high blood pressure, arthritis, heart disease, or cancer?
   1. Yes
   2. No 🡪 skip next Q

1. How many chronic illnesses does the patient have?
   1. 1 or 2
   2. 3 or more
2. The patient’s age
   1. 65-69
   2. 70-74
   3. 75-79
   4. 80+
3. How does the patient describe him/herself?
   1. Male
   2. Female
   3. Non-binary / third gender
   4. Prefer to self-describe ________
   5. Prefer not to say
4. What is the highest grade or level of school that the patient has completed?

a. High school or less

b. Some college or technical school

c. 4-year college degree or some graduate school

d. Masters or doctoral degree

1. What language(s) does the patient usually speak at home? Check all that apply.

a. English

b. Spanish

c. Other

1. Is the patient of Spanish/Hispanic/Latinx ethnicity?
   1. Yes
   2. No
2. What does the patient consider to be his/her racial background? Check all that apply.
   1. American Indian or Pacific Native
   2. Asian
   3. Black or African American
   4. Native Hawaiian or Pacific Islander
   5. White
   6. Other
3. Please write any other comments you would like to make. We truly appreciate your honest feedack.

No word limit

24. This is a research project, and we would like to interview a few care partners by phone. If you would be willing to speak further with one of the researchers about the Health Priorities questionnaire, please write your name and phone number or email address below.

No word limit

Submit button

Your feedback is important.

Thank you very much for answering these questions.
