# Supplementary material for: Leveraging an Electronic Health Record Patient Portal to Help Patients Formulate Their Health Care Goals: Mixed Methods Evaluation of Pilot Interventions
Source: JMIR Form Res. 2024 Aug 29;8:e56332. doi: 10.2196/56332 (PMC11393498; doi:10.2196/56332)
Supplement: Multimedia Appendix 4 [file formative_v8i1e56332_app4.doc]

Clinician group discussion guide

Patient Priorities Care

January 27, 2023

Welcome, introductions, framing 10 min.

1. Development of PPC at Yale (shown effective in face-to-face interviews). Our objective to find a way to make it work with an EMR. First pilot.
2. Importance of candid feedback. Academic paper. May use quotes but won’t identify speaker.
3. Obtain permission to record the session.

Value of the questionnaire 25 min.

1. How useful were patients’ answers to PPC questions? Describe examples that you remember.
2. Did you discuss the patients’ answers with them during their visits? How useful was the discussion? If you did not discuss, why not?
3. Describe any new insights/care changes that you remember that resulted from the PPC questionnaire and discussion.
4. Comments about the questions (that the patients answered)?

Workflow 15 min.

1. Did you read patients’ answers before you started the visit? Was there enough time in the visit to discuss the patients’ priorities?
2. How easy was it to incorporate this discussion into your Epic and visit workflow? How often did you document the discussion in your visit note?
3. Comments about the summary of patients’ answers in Epic? (Show screenshot.)

Bottom line 10 min.

1. Would you like to continue using the PPC questionnaire? Why or why not?
2. Would you advise training clinicians about this tool? (or is it self-explanatory?)
3. Other suggestions for improvement related to Patient Priorities Care?
