# Supplementary material for: Leveraging an Electronic Health Record Patient Portal to Help Patients Formulate Their Health Care Goals: Mixed Methods Evaluation of Pilot Interventions
Source: JMIR Form Res. 2024 Aug 29;8:e56332. doi: 10.2196/56332 (PMC11393498; doi:10.2196/56332)
Supplement: Multimedia Appendix 5 [file formative_v8i1e56332_app5.doc]

**Patient Priorities Interventions using a Portal: 3 Flowcharts**

**Intervention 1a: *MyHealthPriorities* website**

202 patients contacted 🡪 167 did not go to website.

35 went to the website 🡪 2 did not pass registration page.

33 passed registration page 🡪 10 did not answer all Qs.

23 answered all Qs 🡪 13 did not print or save.

10 printed/saved 🡪 5 did not bring to appointment.

5 patients brought Summary to appointment ->

_ _ _ _ _ _ _ _ _ _ _ _ _ _ _ _ _ _ _ _ _ _ _ _ _ _ _ _ _ _ _ _ _ _ _ _ _ _ _ _ _ _ _ _ _ _ _ _ _ _ _ _ _ _ _ _ _ _ _ _ _ _ _ _

**Intervention 1b: Revised *MyHealthPriorities* website**

212 patients contacted 🡪 180 did not go to website.

32 went to the website 🡪 1 did not pass registration page.

31 passed registration page 🡪 12 did not answer all Qs.

19 answered all Qs 🡪 6 did not print or save.

13 printed/saved 🡪 9 did not bring to appointment.

4 patients brought Summary to appointment ->

_ _ _ _ _ _ _ _ _ _ _ _ _ _ _ _ _ _ _ _ _ _ _ _ _ _ _ _ _ _ _ _ _ _ _ _ _ _ _ _ _ _ _ _ _ _ _ _ _ _ _ _ _ _ _ _ _ _ _ _ _ _ _ _

**Intervention 2: EPIC pre-visit questionnaire**

891 patients were sent questionnaires before 1,139 visits 🡪 141 patients kept 0 visits.

750 patients kept >1 visit 🡪552 patients completed 0 Qres.

20 Qres not submitted before visits.

198 completed 205 Qres 🡪 6 Qres submitted before visits not kept.

17 Qres submitted to non-participating nurse

practitioners after changes in the appointments.

153 Qres submitted before kept visits >
